# Language and Color Perception: Evidence From Mongolian and Chinese Speakers

**DOI:** 10.3389/fpsyg.2019.00551

**Published:** 2019-03-14

**Authors:** Hu He, Jie Li, Qianguo Xiao, Songxiu Jiang, Yisheng Yang, Sheng Zhi

**Affiliations:** ^1^College of Educational Science, Inner Mongolia Normal University, Hohhot, China; ^2^Inner Mongolia Autonomous Region Key Laboratory of Psychology, Hohhot, China; ^3^Laboratory of Cognition and Mental Health, Chongqing University of Arts and Sciences, Chongqing, China; ^4^School of Education, Liaocheng University, Liaocheng, China

**Keywords:** language, perception, Mongolian, Chinese, color categorical perception, universal hypothesis, relativistic hypothesis

## Abstract

The present research contributes to the debate in cognitive sentence on the relationship between language and perception by comparing Mongolian and Chinese speakers’ color perception. In this study, featuring a free sorting task and a visual search task comparing Mongolian and Chinese performances, the results show that both universal and relativistic forces are at play. Chinese (Mandarin) and Mongolian color terms divide the blue spectrum differently but the green spectrum, similarly. In Mongolian, light blue (“*qinker*”) and dark blue (“*huhe*”) are strictly distinct, while both light green and dark green are described as one word, *nogvgan*. In Chinese, however, both light blue and dark blue are simply described by one word, *lan*, and both light green and dark green are described by a single word, *lv*. The current study used a free-sorting task and a visual search task to investigate whether this linguistic difference between Chinese and Mongolian speakers leads to a difference in color discrimination. In the free-sorting task, compared with Chinese speakers, Mongolian speakers exhibited different sorting in the blue region (by distinguishing light and dark blue) and the same sorting in the green region. Further results showed that Mongolian speakers discriminated visual search displays that fall into different linguistic categories in Mongolian (e.g., *qinker* or *huhe*) more quickly than visual search displays that belong to the same linguistic category (e.g., both *qinker*) in a visual search task. Moreover, this effect was disrupted in Mongolian participants who performed a secondary task engaging involving verbal working memory (but not a task engaging involving spatial working memory), suggested linguistic interference. Chinese (Mandarin) speakers performing the visual search task did not show such a category advantage under any of the conditions. The finding provides support for the Whorf hypothesis with evidence from an Altay language. Meanwhile, both Chinese and Mongolian speakers reacted faster to the green color than the blue color in the visual search task, suggesting that the variation in human color perception is constrained by certain universal forces. The difference in categorical effects between Chinese and Mongolian speakers in the blue region suggests a relativistic aspect of language and color perception, while the speed of visual search in blue and green suggests a universalistic aspect of language and color perception. Thus, our findings suggest that our perception is shaped by both relativistic and universal forces.

## Introduction

For a long time researchers have debated whether language can affect perceptual experiences, such as color perception. Color categorical perception is faster or more accurate discrimination of colors that straddle a color category boundary, it provides a good opportunity to investigate the basic relationship between language and color perception (Regier and Kay, 2009). Universalists argue that color categorical perception is a universal perceptual effect determined by biological mechanisms and is not language-specific, while relativists posit that color categorical perception is influenced by the categories used to communicate about colors (Regier and Kay, 2009). To our knowledge, most previous studies have focused on Indo-European languages (such as English, Greek and Russian) (Roberson et al., 2005; Gilbert et al., 2006; Winawer et al., 2007; Athanasopoulos et al., 2010), thus broader cross-language comparisons are required to further understand the issue. To fill in this gap, the present research aims to investigate color categorical perception in an Altay language (specifically, Mongolian), hoping to provide more complete information to increase our knowledge of the extent of the influence of linguistic diversity on perception. Lots of previous cross-language studies used between subjects design to compare color categorical perception between different language, however, individual differences in between subjects design can affect the accuracy of the study, thus, the present research use mix design that contain within subjects design to compare color categorical perception between different color, hoping to overcome the influence of individual differences on color category perception.

In a classic study, Berlin and Kay (1969) obtained samples of color areas and category foci-colors (best color exemplars) from native speakers of 20 different languages and found that the color terms and foci are universal. Some studies also concluded that color categories are not language-relative and that foci colors are universal (Witzel and Gegenfurtner, 2011; Yang et al., 2016). However, according to a large number of cross-linguistic studies, color categorical perception is tightly linked to language (Athanasopoulos et al., 2011) and culture (González-Perilli et al., 2017) exposure from birth, supporting the Whorf hypothesis that color categorical perception is language-relative rather than universal.

Regarding the functional organization of color categorical perception in the brain, some results support the Whorf hypothesis and relativistic perspective. Several researchers have deduced that the right visual field is significantly involved in color categorical perception, because the left cerebral hemisphere is preferentially involved in nearly all language tasks, including those requiring lexical access (Gilbert et al., 2006). Gilbert et al. (2006) found that color categorical perception appears in only the right visual field and not in the left visual field in lateralized visual search tasks, implying that color categorical perception is a linguistic phenomenon. Drivonikou et al. (2007) found that significant color categorical perception appears in both visual fields, these results illustrate that both language and perception might affect color categorical perception, however, because more significant effects are observed in the right visual field, the role of language is stronger. Roberson et al. (2008) demonstrated color categorical perception in only the right visual field in certain faster-responding subjects, the result that color categorical perception occurs in only the left hemisphere in faster subjects implies that color categorical perception is a language priority, because the left cerebral hemisphere is preferentially involved in those requiring lexical access (Gilbert et al., 2006). Bird et al. (2014) found that categorical and metric hue differences were coded in qualitatively different ways in different brain regions. Specifically, the middle frontal gyrus responds to categorical color differences but not to hue differences. The middle frontal gyrus is a brain area known for higher cognitive processing, including linguistic processing. In summary, these studies have found that color categorical perception in adults exhibits left brain dominance effect, so they conclude that color categorical perception in adults is a language effect or, at least, a language priority.

Previous cross-language investigations also have revealed that color categorical perception is a linguistic rather than a perceptual phenomenon. Roberson et al. (2005) investigated Himba (a language spoken in Northern Namibia), which contains five color terms. They suggested that Himba speakers have a color perception organization that differs from those speakers of Berinmo, although between languages with broadly similar color categories. Roberson et al. (2008, 2009) tested Korean color terms that do not exist in English and found color categorical perception in only the Korean speakers, despite that both English and Korean speakers share the same discrimination thresholds. Likewise, Winawer et al. (2007) examined Russian speakers’ color perception. There are two terms in Russian distinguishing between light blue (“*goluboy*”) and dark blue (“*siniy*”), whereas there is only one term in English, blue. Russian speakers exhibited the categorical advantage in the rapid color discrimination task using blue stimuli, and this categorical advantage was disrupted by verbal interference and not by spatial interference, this result suggests that the effect of language is online (Roberson and Davidoff, 2000). Using the event-related potential technique, Thierry et al. (2009) observed that the amplitude differences in two color terms in Greek, i.e., *ghalazio* and *ble*, which distinguish light and dark blue, the speakers of Greek led to a greater brain potential amplitude of visual mismatch negativity than English speakers, because there is only one blue color term (blue) in English; and the speakers of Greek demonstrated a greater brain potential amplitude of visual mismatch negativity than distinguish light and dark green, because there is only one green color term (*prasino*) in Greek. The visual mismatch negativity is a marker of an automatic and unconscious process, thus, language-specific categories have an implicit effect on human color perception. These findings on color categorical perception revealed that language modulates ongoing color perception (Lupyan, 2012).

On the other hand, other studies of color categorical perception have shown a different picture. Witzel and Gegenfurtner (2011) found that the color categorical effect might not be robust, even if it existed, and the category effect might be independent of language. Yang et al. (2016) found that colors of different categories are represented differently in the visual cortex of prelinguistic infants, suggesting that categorical color perception does not necessarily depend on language, just as some other areas of perception (Cong et al., 2018). Taken together, the relationship between language and cognition may be more complicated. Franklin et al. (2005, 2008) provided a dynamic perspective, by suggesting that color categorical perception may be universal but shaped by language at a later stage.

**FIGURE A1:**
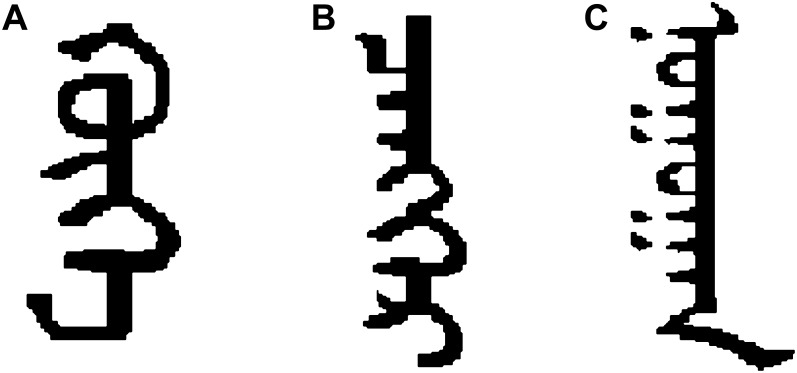
Color terms in Mongolian: **(A)** dark blue (“*Shen* *Lan”* in Chinese, /*huhe*/in Mongolian), **(B)** light blue (“*Qian* *Lan*” in Chinese, /*qinker*/in Mongolian), and **(C)** green (“*lv*” in Chinese, /*nogvgan*/ in Mongolian).

Mongolian, an Altay language, divides the blue region of the color space into a darker shade called *huhe* and a lighter shade called *qinker*, while both lighter green and darker green are described with one word, *nogvgan*, as shown in Appendix Figure A1. In contrast, Mandarin Chinese uses a single word, *lan*, to describe both light blue and dark blue and a single word, *lv*, to describe both light and dark green. In terms of the number of basic color terms in the blue and green region, Mongolian is similar to Russian, Greek or Japanese, whereas Mandarin Chinese is similar to English. Expanding previous research on color perception to speakers of Mongolian and Chinese, two understudied languages, we aimed to examine whether linguistic differences lead to differences in color discrimination between Chinese and Mongolian speakers.

To compare the color categorical perception of Chinese speakers with that of Mongolian speakers, our study investigates Chinese (Mandarin) and Mongolian speakers performing a visual search task using blue stimuli spanning the *qinker/huhe* border and green stimuli, and examines the mechanism of color categorical perception in Mongolian speakers performing a secondary task engaging verbal and nonverbal working memory (Gilbert et al., 2006).

First, to establish linguistic differences in color classification, we presented participants in Experiment 1 a free-sorting task (Roberson et al., 2008). If language affects color categories, Mongolian speakers should divide the dark blue and light blue colors into two categories whereas Chinese speakers should put them into the same category. As both languages have only one term for green, both Chinese and Mongolian speakers should put light and dark green colors into a single category.

Next, if linguistic effects on color perception are specific to the categories encoded in a speaker’s language, Mongolian speakers should be faster in the visual search of a target that falls into a different category from the surrounding colors (e.g., *qinker* surrounded by *huhe*) than the search of a target that falls into the same category as the surrounding colors (e.g., *qinker* surrounded by *qinker*). But this effect should not be observed in the *nogvgan* region because the Mongolian language has only one basic color term for green, *nogvgan*. Likewise, as the Chinese language has only one basic color term for blue (*lan)* and *green (lv)*, respectively, Chinese speakers should not display differences in speed when discriminating dark and light blue colors, or dark and light green colors. Experiment 2 was designed to test these predictions.

Experiment 3 further examined linguistic influences on color perception with interference tasks. If linguistic processing plays an active, online role in color categorical perception (Winawer et al., 2007), the *qinker/huhe* categorical advantage in the Mongolian speakers should be disrupted by verbal, but not nonverbal, interference.

## Materials and Methods

### Experiment 1 Free-Sorting Task

#### Participants

Sixty native Chinese (Mandarin)-speaking undergraduates (24 males and 36 females, mean age 20.9 years) and 54 native Mongolian-speaking undergraduates (21 males and 33 females, mean age 21.1 years) at Inner Mongolia Normal University participated for course credits. All participants were residents from the Inner Mongolia Autonomous Region of China. The sample sizes were determined based on feasibility, and all participants had normal or corrected-to-normal vision based on self-reports.

#### Stimuli

Ten color patches were selected from the green-blue spectral region (1, 2, 3, 4, 5, 6, 7, 8, 9, and 10), as shown in Appendix Table A1. Twenty participants were asked to name these patches by pressing the “0” (*nogvgon* or *lv*), “1” (*qinker* or *lan*), or “2” (*huhe*) key on the computer keyboard. Each color was randomly presented 5 times, resulting in a total of 50 trials. Based on a pretest, three green color patches (3, 6, and 9) were always named “*lv*” by Chinese speakers and “*nogvgan*” by Mongolian speakers. Furthermore, six color patches (1, 2, 4, 5, 7, 8, and 10) were always named “*lan*” (blue) by Chinese speakers and *qinker* (light blue: 1, 2, 5, and 7) and *huhe* (dark blue: 4, 8, and 10) by Mongolian speakers. The color patches measured 20 × 20 mm and were printed on paper. Appendix Table A1 provides the Committee Internationale d’Eclairage (CIELab) coordinates of all stimuli.

#### Procedures

The free-sorting task always preceded color naming to avoid introducing a name grouping bias. The color patches were spread on the paper in random order, and the participant was asked to classify the patches and sort those that appeared similar together, allowing members of the same color family to be grouped. The participants were informed that there was no right or wrong way to complete the task. After the participants completed the sorting task, the groupings were recorded by the experimenter, and the data were processed by the multidimensional scaling method (Roberson and Davies, 2005).

#### Results

**FIGURE 1:**
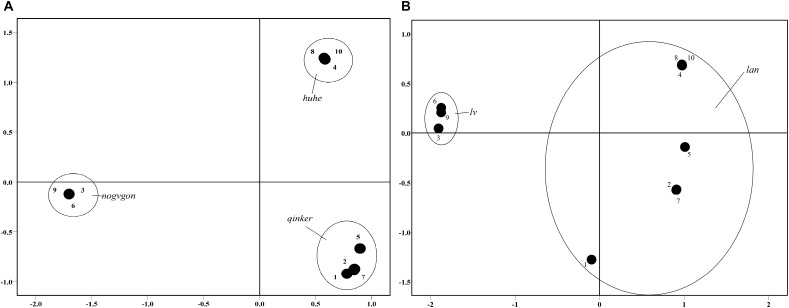
**(A)** Two-dimensional scaling solution for Mongolian free-sorting of the *chagon/qinker/huhe* color patches (Stress = 0.015, RSQ > 0.999). **(B)** Two-dimensional scaling solution for Chinese free-sorting of the *Iv/lan* color patches (Stress = 0.012, RSQ > 0.999).

Figure 1 illustrates the two-dimensional solution for naming task, these patches in Mongolian (Figure 1A) and Chinese (Figure 1B). The results show that the color patches shared the same location when sharing a single color term, such as the green color patches in Chinese (*lv*) and Mongolian (*nogvgan*), or the blue patches in Chinese (*lan*). When the color patches were labeled with two different color terms, however, they were maximally distant, such as the color patches of blue vs. green (*lan* vs. *lv*) in Chinese those of light blue, dark blue and green (*qinker*, *huhe*, and *nogvgan*) in Mongolian.

### Experiment 2 Visual Search Task

#### Participants

Thirty-two native Chinese (Mandarin)-speaking undergraduates (13 males and 19 females, mean age 20.4 years) and thirty-two native Mongolian-speaking undergraduates (15 males and 17 females, mean age 21.1 years) at Inner Mongolia Normal University were paid 10 RMB yuan for their participation. All participants were residents from the Inner Mongolia Autonomous Region of China. The sample sizes were determined based on feasibility, and all participants had normal or corrected-to-normal vision based on self-reports. This study was carried out following the recommendations of the operating guidelines of the Education Science College Ethics Committee (Inner Mongolia Normal University), and written informed consent was obtained from all subjects. All subjects provided written informed consent in accordance with the Declaration of Helsinki. The protocol was approved by the Education Science College Ethics Committee.

#### Stimuli and Apparatus

**FIGURE 2:**
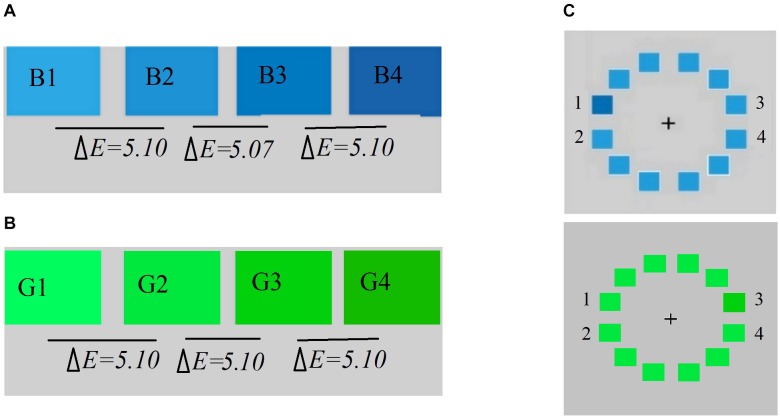
**(A)** Visual search of the blue colors used; B1-B2 and B3-B4 are within-categories, while B2-B3 are between-categories. **(B)** Visual search of the green colors used; G1-G2 and G3-G4 are within-categories, while G2-G3 are between-categories. **(C)** Sample display for the visual search task in which the target occupied any of the four positions (position 1, 2, 3, or 4). Delta E refers to the color difference between the corresponding color stimuli in the CIElab system.

The participants were asked to name the patches by pressing the “0” (*nogvgon* or *lv*), “1” (*qinker* or *lan*), or “2” (*huhe*) key on the computer keyboard. Each color was randomly presented 5 times, resulting in a total of 40 trials. There were four blue color patches, with equal distance in color difference between two adjacent patches, ranging from light blue to dark blue in the CIElab system, as shown in Figure 2A. The pretest established that patches B1 and B2 were named “*qinker*” and the patches B3 and B4 were named “*huhe*” by Mongolian speakers, while all these patches were named “blue” (*lan*) by Chinese speakers. Likewise, four green color with equal distance between two adjacent patches stimuli were presented, from light green to dark green in the Committee Internationale d’Eclairage system, as shown in Figure 2B. The naming task revealed that all of these patches were named “*green*” by both Chinese speakers and Mongolian speakers; Appendix Table A2 provides the Committee Internationale d’Eclairage (CIELab) coordinates of these stimuli.

The stimulus display consisted of a ring of 12 patches surrounding the fixation marker. Color patches measured 1.5 cm × 1.5 cm and were separated by 0.6 cm. All patches were the same color except for one, i.e., the target, as shown in Figure 2C. The target randomly appeared at only one of four positions (position 1, 2, 3, or 4) (Zhou et al., 2010). The target and distractor colors were either from different lexical categories (e.g., different shades of *qinker*, B2-B3; G2-G3) or from the same lexical category (e.g., a *qinker* and a *huhe*, B1-B2 or B3-B4; G1-G2; or G3-G4). Although four blue patches are within categories in Chinese, and four green patches are within categories in Chinese and Mongolian, to facilitate a comparison with Mongolian *qinker/huhe*, this experiment proceeded as if both within and between categories existed for these colors (Winawer et al., 2007; Roberson et al., 2008).

#### Procedures

In the visual search task, the participants sat approximately 600 mm from the screen in a soundproof room lit by an 8W fluorescent lamp. The light intensity in the room was approximately 100 lux, and the screen had a 17-inch LCD display with a resolution of 1600 × 1280, a refresh rate of 60 Hz, and a brightness of 50%, sharpness of 50%, and color saturation of 100%. Each trial began with the onset of a central fixation marker for 500 ms on a neutral gray screen, such that the red-green-blue (RGB) values were 192, 192, and 192. Then the stimulus display appeared, consisting of a ring of 12 squares surrounding the fixation marker. Participants’ task was to indicate whether the target – the unique color patch – was on the left or right half of the circle (Zhou et al., 2010; Zhong et al., 2015), by pressing one of two keys on the keyboard using either the left (“F” key) or right (“J” key) index finger. The instructions emphasized that the participants should respond as quickly and accurately as possible to the visual search displays within 2500 ms. After each response, the screen was blank for 500 ms before the start of the following trial. After a practice block of 16 trials, each participant completed 64 trials in the visual task. The study followed a 2 (speakers: Chinese vs. Mongolian) × 2 (categorical type: within or between category) × 2 (color: blue vs. green) design, with the latter two variables as within-participant factors.

#### Results

**FIGURE 3:**
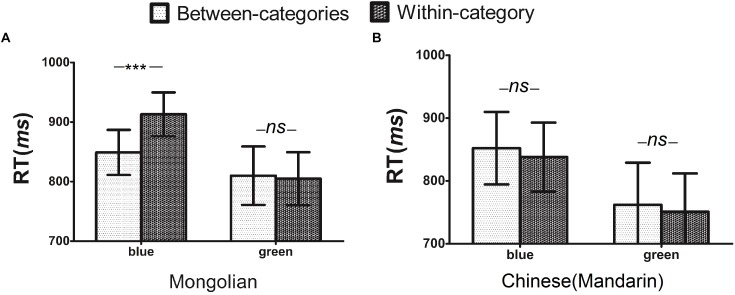
For the visual search task, **(A)** a color categorical perception (CCP) for blue compared with green was shown in Mongolian, while **(B)** a CCP was not shown in Chinese (Mandarin). Error bars represent the mean with a 95% CI, ^∗∗∗^*p* < 0.001, ANOVA, *df* = 62; *ns*, nonsignificant.

As plenty of time was given to participants, the overall accuracy was high (94.2% for the Chinese speakers and 94.0% for the Mongolian speakers). No trade-off between speed and accuracy was observed, thus, our analysis focused on the reaction time. Trials in which a participant pressed the wrong key or the reaction time was greater than 2 standard deviations from the participant’s mean were not included in the analysis of the visual search data. As a result, approximately 8.3% of all trials were excluded, among which 87.5% were due to erroneous responses. The reaction time data from the search task were analyzed with a 2 (speakers: Chinese vs. Mongolian) × 2 (categorical type: within- *vs.* between) × 2 (color: green vs. blue) analysis of variance, with the latter two factors as within-participant factors. The three-way interaction was significant [*F*(1, 62) = 18.81, *p* < 0.001, η^2^ = 0.23], and the interaction of categorical type × color was significant [*F*(1, 62) = 16.23, *p* < 0.001, η^2^ = 0.21]. The interaction of categorical type × speakers was also significant [*F*(1, 62) = 18.51, *p* < 0.001, η^2^ = 0.23]. As seen in Figure 3, Mongolian speakers were faster at identifying between-category than within-category stimuli (between-categories: 849 ± 105 ms vs. within-categories: 913 ± 102 ms), for the color of blue, but not for the color of green (between-categories: 810 ± 137 ms vs. within-categories: 806 ± 123 ms). As expected, Chinese speakers showed no difference in reaction time for identifying green (between-categories: 763 ± 127 ms vs. within-categories: 752 ± 116 ms) or blue colors (between-categories: 852 ± 121 ms vs. within-categories: 838 ± 114 ms). Notably, there were some unexpected results, such as reaction time was faster for the green color (783 ± 123 ms) than the blue color (863 ± 107 ms), *F*(1, 62) = 45.53, *p* < 0.001, η^2^ = 0.42; Mongolian speakers were faster at discriminating *nogvgan* colors than discriminating the *qinker/huhe* pairs, *F*(1, 62) = 4.51, *p* < 0.05, η^2^ = 0.09. The present result showed that linguistic effects on color perception were specific to the categories encoded in a speaker’s language, and next experiment would examine the mechanism of linguistic effects on color perception.

### Experiment 3 Interference Task

#### Participants

Forty native Mongolian-speaking undergraduates from Inner Mongolia Normal University in Inner Mongolia (16 males and 24 females, mean age 20.9 years) were paid 15 RMB yuan for their participation. All participants were from Inner Mongolia Normal University and originated from the Inner Mongolia Autonomous Region of China. The sample sizes were determined based on feasibility, and all participants had normal or corrected-to-normal vision based on self-reports. The ethics aspect reports were the same as those reported for Experiment 2.

#### Stimuli, Apparatus, and Procedure

**FIGURE 4:**
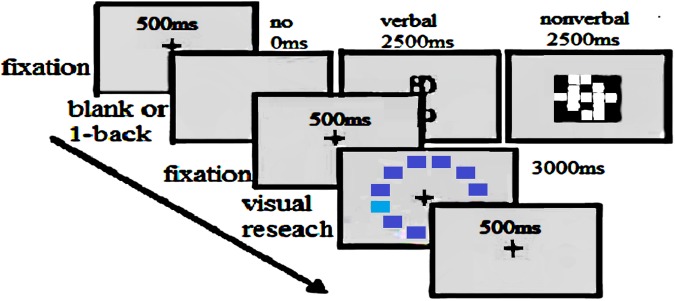
Trial events. Within a block of trials, the visual search task was interwoven with blank displays, displays containing a color word, or displays containing a spatial grid.

Only blue color patches, used were the same as those used in experiment 2. In this experiment, each participant completed the following three tasks after a practice block of 8 trials: visual search under no-interference (64 trials), verbal-interference (32 trials), and nonverbal-interference (32 trials) conditions. The order of the three tasks was counterbalanced across the participants, as Figure 4.

The visual search and interference stimuli were interleaved (Figure 4). The lab and screen were the same as the visual search task in Study 2. As shown in Figure 4, A fixation marker was presented for 500 ms. The fixation marker was then replaced by a blank screen (no-interference), a Mongolian color term (verbal-interference), or one of the spatial grids (nonverbal-interference) for 2,500 ms. In the verbal-interference task, the interference display consisted of a Mongolian color word drawn from the following set: “*hara* (black),” “*cagan* (white),” “*unesu* (gray),” “*ulabur* (orange),” “*boro* (purple),” “*ulagan* (red),” “*nogvgan* (green),” “*hureng* (brown),” and “*sira* (yellow)” (Gilbert et al., 2006). In the nonverbal-interference task, the displays consisted of a 5 cm × 5 cm grid in which 12 of the 25 squares were black and 13 were white in a set of 6 displays. After the visual search task, the participants were required to press the space bar with both thumbs whenever they detected that the secondary task stimulus was the same as that shown in the previous display (one-back match) (Gilbert et al., 2006).

#### Results

**FIGURE 5:**
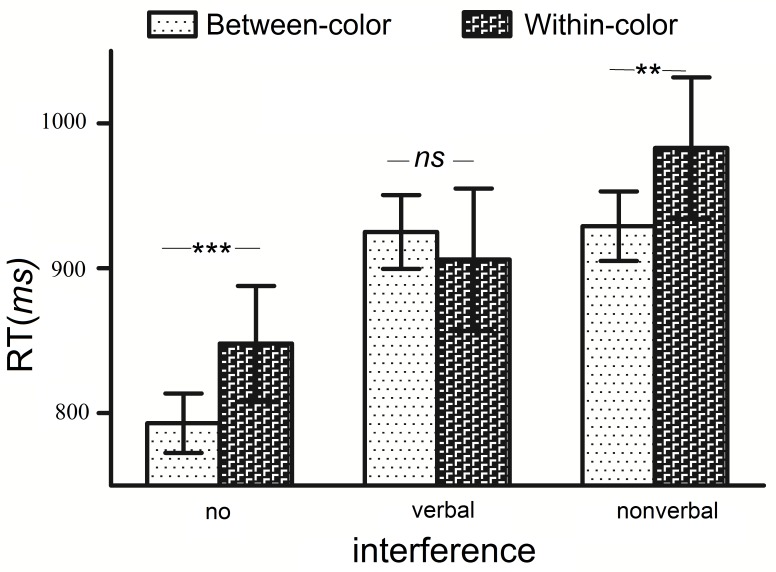
Mongolian speakers’ reaction times under the no-interference, verbal-interference, and nonverbal-interference conditions. The no-interference results replicated those obtained in experiment 2. Under the nonverbal-interference condition, the performance followed a pattern similar to that observed in the no-interference condition. *^∗∗^P* < 0.01, ^∗∗∗^*p* < 0.001, ANOVA, *df* = 35; *ns*, nonsignificant. Error bars represent a 95% CI.

Two participants were excluded because their mean reaction times were greater than 2 standard deviations from the group mean reaction time, and another two participants were excluded because their accuracy was lower than 80% in the visual task or one-back match task. After excluding those participants, the accuracy in the visual task was 90.1%, and the accuracy in the one-back match task was 92.1%. No trade-off between speed and accuracy was observed, thus, our analysis focused on the reaction time. Trials in which a participant pressed the wrong key or the reaction time was greater than 2 standard deviations from the participant’s mean were not included in the analysis of the visual search data. Approximately 16.3% of all trials were excluded using the abovementioned criteria, and 87.5% of these trials were excluded due to erroneous responses. To directly compare the no-interference and interference conditions, we analyzed the Mongolian participants’ reaction time data and performed a 2 (pair type; within- vs. between-categories) × 3 (interference: no- vs. verbal- vs. nonverbal-interference) analysis of variance. Significant main effect of pair type [*F*(1, 35) = 11.44, *p* < 0.01, η^2^ = 0.25] and interference [*F*(2, 70) = 33.90, *p* < 0.001, η^2^ = 0.49] were observed, and the interaction was significant [*F*(2, 70) = 5.76, *p* < 0.01, η^2^ = 0.14]. The simple main effects are shown in Figure 5, The reaction time data from the search task under the no-interference and nonverbal-interference conditions were similar to those observed in Experiment 2, Mongolian speakers were faster at between-category than within-category judgments, however, color categorical perception was disrupted by verbal interference, as shown in Figure 5. The simple main effect of pair type was significant in the [no-interference [(between-categories: 793 ± 123 ms vs. within-categories: 848 ± 118 ms): *F*(1, 35) = 15.4, *p* < 0.001, η^2^ = 0.31] and; nonverbal-interference [(between-categories: 929 ± 144 ms vs. within-categories: 983 ± 175 ms): *F*(1, 35) = 7.79, *p* < 0.01, η^2^ = 0.18] conditions.

## Discussion

Numerous cross-linguistic investigations of color categorization have provided abundant evidence regarding the relationship between language and perception (Winawer et al., 2007; Roberson et al., 2008; Regier and Kay, 2009; Thierry et al., 2009). The present investigation compared blue or green discrimination between Chinese and Mongolian speakers. The current results demonstrate that both Mongolian and Chinese participants are faster at discriminating green, which is consistent with the Spectral Luminous Efficiency Function, revealing that humans have the highest resolution of green light in photopic vision (De Vries, 1946). The Spectral Luminous Efficiency Function is rooted in retinal effects (Bieber et al., 1998) and derives from natural selection during the course of evolution, suggesting that some aspects of human color perception are universal. Lots of studies exploring color categorical perception support the Whorf hypothesis that human cognition is influenced by language and culture. Numerous previous studies partially support the Sapir-Whorf hypothesis and suggest that the relativistic perspective is constrained by universal forces. These studies demonstrate that on the one hand, perceptual processes require a physiological basis, but on the other hand, language is a powerful tool for modulating perception across development (Franklin et al., 2005, 2008) in a flexible and task-dependent manner (Lupyan and Clark, 2015).

In the first experiment, we compared the color performance of the Mongolian speakers and Chinese speakers in a free-sorting task. Compared with the Chinese speakers, the Mongolian speakers sorted the blue patches differently and sorted the green patches the same sort, however, in a within-subject design, the Mongolian speakers exhibited different sorting patterns between the green and blue patches. The free-sorting of the Mongolian speakers was similar to that of Russian speakers in the free-sorting task (Roberson and Davies, 2005), and Russians have two color terms for the blue patches but one color term for the green patches. Furthermore, the Chinese speakers showed a free-sorting performance similar to that of English speakers in the free-sorting task (Roberson and Davies, 2005), and both of these languages have one color term for both the blue and green patches. Thus, the number of basic color terms leads to different performances in the color free-sorting task, and the basic color term leads to color categories.

The second experiment investigated Chinese and Mongolian speakers performing a visual search task using language as a within subjects variable to compare color categorical perception between blue and green color stimuli. This result overcomes the influence of individual differences, and it is consistent with the Sapir-Whorf hypothesis, which suggests that different lexical codes for color may induce differences in color perception (Zhong et al., 2015). This finding was consistent with the data from Russian, Greek (Thierry et al., 2009) and Japanese (Athanasopoulos et al., 2011) speakers in the blue region and Korean speakers in the green region (Roberson et al., 2008). Many previous studies investigating color categorical perception proposed that the mechanism of color categorical perception is related to the postnatal language processing involved in color perception, particularly, verbal-coded colors influence color discrimination (Liu et al., 2009; Liu et al., 2010). In the current study, the different color categorical perceptions between the Mongolian and Chinese speakers suggests that color vocabulary may influence the coding of color vision. González-Perilli et al. (2017) suggested that culture may also affect color categorical perception and that some nonverbal experiences may shape color categorical perception, which is a worthwhile question for future research to explore.

However, the visual search task found that green elicited faster visual research reaction times than blue in both Mongolian speakers and Chinese speakers, consistent with the Spectral Luminous Efficiency Function (De Vries, 1946), this result implies the extent of the influence of linguistic diversity on perception is biologically constrained (Skelton et al., 2017), suggesting that the relativistic perspective of human color perception is constrained by universal forces. The predictive processing hypothesis (Lupyan and Clark, 2015) and the Bayesian model (Tajima et al., 2016) reflect an interplay between downward-flowing predictions and upward-flowing sensory signals. The sensory signals may link the universal forces, while downward-flowing predictions may link some Whorfican forces (such as culture or language). Thus, the linguistic relativity is modulated by some universal factor, some details of the modulating process need further researched.

Witzel and Gegenfurtner (2013) found that color categorical effects were sensitivity to color differences, and observe that only JNDs of unsaturated green were lower than blue in the spatial 4-Alternative Forced-Choice (4AFC) discrimination task, it implying that sensitivity for color differences should be related to the characteristics of the color itself. Thus, color perception studies should equalize the chromatic aberration of color stimuli from multiple channels, such as Munsell system (Franklin et al., 2005; Gilbert et al., 2006), Derrington-Krauskopf-Lennie (DKL) color space (Witzel and Gegenfurtner, 2013, 2018). The present study equalizes the chromatic aberration of stimuli in the Committee Internationale d’ Eclairage (CIELab) space. Many previous studies did not control color rendering, the studies of Witzel and Gegenfurtner declared that the color rendering might impact on color categorical effects, thus, we should pay attention to equalize color rendering in future color categorical perception research. Lupyan and Clark (2015) suggested that language can affect perceptual experiences in a flexible and task-dependent manner, it is also necessary to aggregate color categorical effects research results from different perspectives through more tasks in the future, such as attentional-blink paradigm (Maier and Abdel Rahman, 2018), oddball (Thierry et al., 2009), and just-noticeable differences testing (Witzel and Gegenfurtner, 2015).

The result of the third experiment not only replicated the Mongolian speakers’ color categorical perception but also investigated its mechanism. The findings from the interference task suggest that the effect was disruptive in participants performing a secondary task that engaged verbal working memory but not in a task that engaged nonverbal working memory, supporting a notion suggested by Winawer et al. (2007); Roberson and Davidoff (2000), and Gilbert et al. (2006) that color categorical perception is an online language effect. This result is consistent with the conclusions drawn by Winawer et al. (2007); Roberson and Davidoff (2000), and Gilbert et al. (2006) who suggest that color categorical perception is disrupted by verbal interference but not by nonverbal interference. The present result suggests that language effect in color categorical perception arise as a function of the interaction of lower-level perceptual processing and higher-level knowledge systems (e.g., language) online. Some event-related potential studies found that the color categorical effect of visual mismatch negativity (Thierry et al., 2009) and P1 (Maier and Abdel Rahman, 2018), because both visual mismatch negativity and P1 are the marker of an automatic and unconscious process, those result suggested that language-specific categories have an automatic and unconscious effect on human color perception.

The relationship between language and perception has constantly been a classic debate, and consensus has swung back and forth between these two poles over the years (Regier and Kay, 2009). At one pole of this debate is the relativist perspective, which states that our perception is shaped by the semantic categories of our native language, and some studies exploring color categorical perception support the relativist perspective (Gilbert et al., 2006; Winawer et al., 2007). At the other pole is the universal perspective, which states that a universal repertoire of perception exists that leaves its imprint on languages (Franklin et al., 2005; Regier and Kay, 2009; Witzel and Gegenfurtner, 2011; Yang et al., 2016), support this universal perspective. In recent years, the relationship between language and perception has been better elucidated by the rejection of distinguishing between language and perception representations, and adopting more complex perspective between universalism and relativism, such as the predictive processing hypothesis (Lupyan and Clark, 2015), Bayesian model (Tajima et al., 2016), and investigations of development (Franklin et al., 2005, 2008). These complex perspectives imply that perception is shaped by both relativistic and universal forces, and our parallel findings obtained using two very different manipulations, i.e., a cross-linguistic comparison and an interference task, converge to provide support for these complex perspectives in some eastern language.

## Conclusion

In conclusion, the present research provides insight into how Chinese and Mongolian speakers spontaneously categorize and discriminate colors, and suggests that the basic relationship between language and cognition may be complex and that perception may be shaped by both relativistic and universal forces.

## Author Contributions

HH, YY, and JL designed the experiments. HH, JL, SJ, and SZ carried out the experiments. HH and JL analyzed the experimental results. HH, JL, and QX wrote the manuscript.

## Conflict of Interest Statement

The authors declare that the research was conducted in the absence of any commercial or financial relationships that could be construed as a potential conflict of interest.

## Appendix

**Table A1:** CIELAB coordinates of the stimuli crossing the boundary in the free-sorting task.

Color	R	G	B	L	a	B	*E*	Mongolian Name	Chinese Name
1	36	206	210	75.56	-38.22	-13.64	85.76	*qinker*	*lan lv*
2	42	161	218	62.45	-12.34	-38.26	74.27	*qinker*	*Lan*
3	70	221	165	79.46	-52.50	16.17	96.60	*nogvgan*	*Lv*
4	4	118	185	47.60	-2.26	-42.84	64.08	*huhe*	*Lan*
5	28	139	203	55.10	-6.64	-41.26	69.16	*qinker*	*Lan*
6	22	169	60	60.67	-58.28	44.94	95.37	*nogvgan*	*Lv*
7	76	184	233	70.66	-15.76	-33.50	79.77	*qinker*	*Lan*
8	26	96	165	39.97	4.35	-43.16	58.98	*huhe*	*Lan*
9	33	177	123	64.32	-49.19	17.51	82.84	*nogvgan*	*Lv*
10	22	79	141	33.32	5.94	-39.47	51.99	*huhe*	*Lan*

**Table A2:** CIELAB coordinates of the stimuli of blue and green.

Color	R	G	B	L	a	b	*E*	Mongolian Name	Chinese Name
B1	42	161	218	62.45	-12.34	-38.26	74.25	*qinker*	*lan*
B2	28	139	203	55.10	-6.64	-41.26	60.16	*qinker*	*lan*
B3	4	118	185	47.60	-2.25	-42.84	64.08	*huhe*	*lan*
B4	26	96	165	39.97	4.35	-43.16	58.98	*huhe*	*lan*
G1	62	255	120	88.88	-73.84	50.87	126.25	*nogvgan*	*lv*
G2	31	234	101	82	-73	51.2	121.14	*nogvgon*	*lv*
G3	0	215	84	75	-72	51	116	*nogvgon*	*lv*
G4	0	194	67	68.22	-71.7	50.1	111	*nogvgon*	*lv*
